# Long-term clinical outcomes after upgrade to resynchronization therapy: A propensity score–matched analysis

**DOI:** 10.1016/j.hroo.2021.06.009

**Published:** 2021-12-17

**Authors:** Mariana Brandão, João Gonçalves Almeida, Paulo Fonseca, Joel Monteiro, Elisabeth Santos, Filipa Rosas, José Nogueira Ribeiro, Marco Oliveira, Helena Gonçalves, João Primo, Ricardo Fontes-Carvalho

**Affiliations:** ∗ Cardiology Department, Centro Hospitalar de Vila Nova de Gaia, Vila Nova de Gaia, Portugal; † Cardiology Department, Centro Hospitalar do Tâmega e Sousa, Penafiel, Portugal

**Keywords:** Cardiac resynchronization therapy, Upgrade, Heart failure, Pacemaker, Implantable cardioverter-defibrillator

## Abstract

**Background:**

Upgrade to cardiac resynchronization therapy (CRT) is common in Europe, despite little and conflicting evidence.

**Objective:**

To compare long-term clinical outcomes in a cohort of patients receiving de novo or upgrade to CRT.

**Methods:**

Single-center retrospective study of 295 consecutive patients submitted to CRT implantation between 2007 and 2018. Upgraded and de novo patients complying with a dedicated follow-up protocol were compared in terms of clinical (NYHA class improvement without major adverse cardiac events [MACE] in the first year of follow-up) and echocardiographic (left ventricle end-systolic volume reduction of >15% during the first year) response.

**Results:**

No differences in the rate of clinical (59.3% vs 62.6%, *P* = .765) or echocardiographic response (72.2% vs 71.9%, *P* = .970) between groups were observed. Device-related complications were also comparable between groups (8.9% vs 8.4%, *P* = .892). Occurrence of MACE and all-cause mortality were analyzed over a median follow-up of 3 (interquartile range 1–6) years: MACE occurred less frequently in the de novo group (hazard ratio [HR]: 0.55, 95% confidence interval [CI]: 0.34–0.90, *P* = .018), but all-cause mortality was similar among groups (HR: 0.87, 95% CI: 0.46–1.64, *P* = .684). Propensity score–matching analysis was performed to adjust for possible confounder variables. In the propensity-matched samples, all-cause mortality (HR: 1.26, 95% CI: 0.56–2.77, *P* = .557) and MACE (HR: 0.84, 95% CI: 0.46–1.54, *P* = .574) were comparable between upgrade and de novo patients.

**Conclusion:**

Survival after upgrade to resynchronization therapy was comparable to de novo implants. Additionally, clinical and echocardiographic response to CRT in upgraded patients were similar to de novo patients.

**Key Findings**

▪ Despite the absence of evidence from randomized trials, upgrade to cardiac resynchronization therapy (CRT) is common and accounted for nearly 30% of implants in the European CRT Survey.
▪ Despite older age and a higher comorbidity burden, upgraded patients exhibited similar clinical and echocardiographic response to CRT to de novo patients.
▪ Survival after upgrade to resynchronization therapy was comparable to de novo implants.
▪ Upgrade to CRT may confer morbidity and mortality benefits to patients with heart failure, as previously shown in randomized trials with de novo device recipients

## Introduction

The unequivocal benefit of cardiac resynchronization therapy (CRT) in appropriately selected patients with heart failure (HF) with reduced ejection fraction is well established. The landmark randomized controlled trials have demonstrated clinical improvement and significant reductions in morbidity and mortality^1^, ^2^, ^3^ with de novo CRT.

Adverse remodeling and worsening of left ventricular (LV) function following right ventricular (RV) pacing has been demonstrated. HF due to mechanical dyssynchrony occurs in up to 50% of pacemaker recipients.^4^ However, in certain cases, whether this is attributable to pacing or represents natural disease progression is often difficult to discern.

Upgrade to CRT from previous conventional pacemaker or implantable cardioverter-defibrillator (ICD) is common practice among European countries. In fact, upgrades accounted for 29.2% of CRT implants in the European CRT Survey,^5^ despite the absence of evidence from large randomized trials. Patients with previous devices were, however, excluded from the major CRT trials.

Patients with previous standard indications for pacemaker who subsequently develop HF symptoms and deterioration of cardiac function and present with high rates of ventricular pacing are often upgraded to CRT, either electively or at the time of generator replacement. ICD recipients who later develop left bundle branch block (LBBB) or bradyarrhythmia with indication for pacemaker also occasionally receive upgrade to CRT.

Interestingly, guidelines are discordant regarding upgrade recommendations. While European Society of Cardiology (ESC) 2013 Pacing and CRT guidelines^6^ provide a class I (level of evidence B) indication, the 2016 ESC HF guidelines^7^ restrict to a class IIb (level of evidence B) recommendation.

Results from published series are also conflicting. Previous studies have shown worse outcomes in upgraded patients.^8^ Contrastingly, others have shown similar benefit in terms of clinical and echocardiographic response and reverse remodeling following upgrade.^9^, ^10^, ^11^ Most of the available data come from patients with previous pacemakers, with even less evidence on upgrade from ICD devices.

In the present study, we have compared the short- and long-term clinical outcomes of patients undergoing upgrade from pacemaker or ICD with those receiving de novo CRT.

## Methods

### Study population

This was a single-center retrospective observational cohort study including all patients consecutively submitted to CRT implantation between 2007 and 2018 in a Portuguese tertiary center.

Baseline characteristics, echocardiographic parameters, implantation, and outcome data were collected to a database.

Patients meeting criteria for CRT implantation had HF with reduced ejection fraction (according to the guidelines in force at the time: 2009 and 2016 ESC HF Guidelines) and QRS width ≥120 ms, and were in NYHA class II–IV.

Except for cases of contraindication or intolerance, patients were under optimal medical therapy prior to device implantation, including renin-angiotensin system inhibitors, beta blockers, and mineralocorticoid receptor antagonists.

Upgrade recipients had a previous pacemaker or an ICD, implanted for conventional bradycardia indications or sudden cardiac death (SCD) prevention, respectively.

Data regarding QRS morphology and duration, LV function, and clinical status prior to the original device implantation were collected when possible. Information derived from previous device interrogation was also reviewed.

The study was conducted in accordance with the Declaration of Helsinki and was approved by the local Ethics Committee.

### Device implantation

CRT implantation was performed according to standard protocols, under local anesthesia. Devices from multiple manufacturers were used (St. Jude Medical/Abbot; Medtronic; Boston Scientific; LivaNova/Microport; Biotronik).

Electrophysiology catheter–facilitated coronary sinus cannulation with electrogram guidance was performed, followed by coronary sinus venography for target vein selection. The LV lead was subsequently implanted in the most suitable side branch, preferably in a posterolateral or lateral location, whenever possible. An epicardial lead was placed in cases of unsuccessful coronary sinus cannulation, in a deferred procedure.

For upgrade procedures, in cases of right-sided previous device, venography was performed to assess patency of the venous system. When right-sided implantation of the LV lead was deemed unsuitable, left-sided cannulation followed by lead tunneling was performed under conscious sedation.

The choice for CRT-pacemaker or CRT-defibrillator (CRT-D) was discussed among the electrophysiology and pacing laboratory team and the patient’s attending cardiologist. The decision of adding back-up defibrillator for primary prevention of SCD was based on performance status, comorbidities, and life expectancy of the patient, as well as the type of cardiomyopathy and cardiac magnetic resonance imaging data, when available. Standard secondary prevention indications were taken into consideration whenever present.

In patients with permanent atrial fibrillation (AF), atrial port plugs were used, and the device was programmed to VVI mode. Concomitant atrioventricular (AV) junction ablation was performed in patients with high ventricular rate refractory to pharmacological treatment.

### Echocardiographic evaluation

All patients underwent baseline echocardiographic evaluation prior to CRT implantation. Standard parameters, including left atrium dimensions, measurements of LV and RV dimensions and function, pulmonary artery systolic pressure, and degree of mitral regurgitation, were recorded whenever available. Mitral regurgitation was assessed qualitatively and graded on a scale of minimal to mild to severe.

Echocardiograms were performed at least twice in the first year of follow-up.

### Follow-up and device optimization

Patients complied with a follow-up protocol that included echocardiographic evaluation and a specific device outpatient program. Patients provided informed consent for data collection in each evaluation. In deceased patients, clinical data refers to the latest available follow-up.

A dedicated device follow-up program was implemented by cardiopulmonary technicians and supervised by the electrophysiology and pacing laboratory medical team. An echocardiographic protocol for device optimization was also employed.

The first device evaluation was scheduled 2 months after the implant. Device interrogation with evaluation of biventricular pacing percentage and arrhythmia burden, and screening for complications (eg, lead dislodgement), were performed. In patients meeting criteria to proceed with the optimization protocol, echocardiographic optimization was scheduled at 3, 6, and 12 months after CRT implant, and at 6-month intervals thereafter. Transmitral Doppler-directed optimization of AV delay (for AV synchrony) and VV delay (for interventricular and intraventricular synchrony) were performed. Patients were discharged from the optimization protocol if they presented with stable echocardiographic parameters over a 12-month period or with recovered LV function, or with minimal residual asynchrony.

However, evidence showing an absence of benefit in routine use of optimization later emerged, and automatic AV-VV optimization algorithms were introduced for most devices. Therefore, echocardiographic optimization was since reserved to symptomatic nonresponders and recipients of devices without automatic optimization algorithms. The previously described schedule was applied in such cases.

### Study endpoints

Outcome measures were clinical and echocardiographic response to CRT, major adverse cardiac events (MACE), and all-cause mortality. MACE included hospitalization for HF or all-cause mortality. Clinical response was defined as NYHA class improvement (at least 1 class), in the absence of MACE in the first year of follow-up. A reduction of more than 15% in left ventricular end-systolic volume (LVESV) in the first year denoted echocardiographic response. Super-response was defined as recovery of LV function, with left ventricular ejection fraction (LVEF) >50% during the first year of follow-up.

### Statistics

Descriptive data were summarized using the appropriate statistical tools, given the nature of the variables involved. Kolmogorov-Smirnov test was used to assess the normal distribution of continuous data. Student *t* test or its nonparametric equivalent (Mann-Whitney *U* test or Wilcoxon signed rank test) were used to compare the distribution of continuous variables. The Pearson χ^2^ test was used to test the association between categorical variables. Survival analysis was performed with Kaplan-Meier method and log-rank test.

Propensity score–matching (PSM) analysis was performed to adjust for possible confounder variables, using the PSM package of SPSS. Both therapy groups (de novo and upgrade) were analyzed for heterogeneity within the distribution of their covariates (Mann-Whitney *U* tests and the χ^2^ test). The model contained covariates with *P* value < .1: age, sex, device type, arterial hypertension, coronary artery disease, AF, valvular heart disease (moderate-to-severe), chronic kidney disease, and therapy with mineralocorticoid receptor antagonists. Caliper matching with a score match tolerance (precision) of 0.05, without replacement, was performed. After propensity score adjustment, both groups were again checked for heterogeneity in their covariates.

A two-sided p-value <0.05 was considered significant. Statistical analysis was performed using 18 SPSS 26 (IBM SPSS, Armonk, New York, USA) software.

## Results

### Baseline characteristics

A total of 295 patients (70.5% male, mean age 67.3±10.7 years) were included in this analysis, of whom 239 (81%) underwent a *de novo* implantation. Fifty-six patients (19%) were upgraded from a prior device.

Upgrade and *de novo* groups were comparable in terms of gender, type of cardiomyopathy, baseline NYHA class and LVEF, and use of disease-modifying therapy and diuretics. Upgraded patients were older (70.0 ± 9.6 vs 66.7 ± 10.8 years, *P* = .034) and had higher rates of AF (58.2% vs 26.7%, *p* < .001), coronary artery disease (41.8% vs 26.2%, *P* = .033), moderate-to-severe valve disease (42.9% vs 22.6%, *P* = .003), and chronic kidney disease (36.4% vs 18.7%, *P* = .008). Patients in the upgrade group were more likely to receive a CRT-pacemaker (71.4% vs 39.3%, *P* < .001) and, when receiving CRT-D, these were more often implanted for secondary prevention (53.3% vs 20.2%, *P* = .011).

LBBB (or paced QRS) was the predominant QRS morphology in the baseline electrocardiogram in both groups (*P* = .084). Echocardiographic parameters were similar between groups. Upgraded patients had higher baseline N-terminal pro-B-type natriuretic peptide levels (*P* = .01). Baseline characteristics of CRT recipients are disclosed in Table 1.

**Table 1:** Baseline characteristics

	All (N = 295)	De novo (N = 239)	Upgrade (N = 56)	*P* value
Male, n (%)	208 (70.5%)	163 (68.2%)	45 (80.4%)	.073
Age (years)	67.3 ± 10.7	66.7 ± 10.8	70.0 ± 9.6	.036
Device type, n (%)				
CRT-P	134 (45.4%)	94 (39.3%)	40 (71.4%)	<.001
CRT-D	161 (54.6%)	145 (60.7%)	16 (28.6%)	<.001
Primary prevention,† n (%)	110 (76.4%)	103 (79.8%)	7 (46.7%)	.011
Type of cardiomyopathy, n (%)				
Ischemic	79 (27.5%)	61 (26.4%)	18 (32.1%)	.487
Nonischemic	208 (72.5%)	170 (73.6%)	38 (67.9%)	
Baseline NYHA class				
I/II	67 (23.3%)	55 (23.7%)	12 (21.4%)	.852
III/IV	221 (76.7%)	177 (76.3%)	44 (78.6%)	
Comorbidities				
Arterial hypertension, n (%)	193 (65.9%)	151 (63.4%)	42 (76.4%)	.096
Diabetes mellitus, n (%)	98 (33.6%)	74 (31.25%)	24 (43.6%)	.110
Coronary artery disease, n (%)	84 (29.2%)	61 (26.2%)	23 (41.8%)	.033
Atrial fibrillation, n (%)	95 (32.6%)	63 (26.7%)	32 (58.2%)	<.001
Valvular heart disease (moderate-to-severe), n (%)	79 (26.8%)	54 (22.6%)	24 (42.9%)	.003
Chronic kidney disease (eGFR <60 mL/min/1.73 m^2^), n (%)	64 (22.1%)	44 (18.7%)	20 (36.4%)	.008
NT-pro-BNP (pg/mL)	3167.0 ± 4303.0	3460.0 ± 4789.2	7444.0 ± 11,946.9	.01
Serum creatinine (mg/dl)	1.2 ± 0.3	1.1 ± 0.5	1.2 ± 0.4	.215
Guideline-directed medical therapy				
ACEI/ARB/ARNI, n (%)	242 (86.4%)	199 (87.7%)	43 (81.1%)	.304
Beta blocker, n (%)	234 (83.6%)	188 (82.8%)	46 (83.6%)	.482
MRA, n (%)	158 (56.4%)	122 (53.7%)	36 (67.9%)	.085
Loop diuretic, n (%)	223 (82.0%)	177 (80.1%)	46 (90.2%)	.136
Baseline electrocardiogram				
Sinus rhythm, n (%)	209 (70.8%)	182 (81.3%)	27 (49.1%)	<.001
Atrial fibrillation, n (%)	60 (21.5%)	38 (17.0%)	22 (40.0%)	<.001
LBBB or paced QRS, n (%)	270 (91.5%)	215 (90.0%)	55 (98.2%)	.084
QRS duration (ms)	170.6 ± 21.5	166.5 ± 18.6	185.2 ± 25.1	<.001
Baseline echocardiogram				
LVEF, %	26.9 ± 6.8	28.8 ± 7.2	28.0 ± 6.4	.357
LVESV (mL)	154.8 ± 68.9	151.5 ± 65.8	165.1 ± 78.0	.225
LA diameter (mm)	47.9 ± 11.5	46.0 ± 7.5	49.3 ± 9.5	.038
Degree of mitral regurgitation, n (%)				
Mild-to-moderate	174 (69.8%)	136 (69.4%)	38 (71.7%)	.260
Moderate-to-severe	75 (30.1%)	60 (30.6%)	15 (28.3%)	
S’ (cm/s)	10.5 ± 3.2	10.6 ± 4.0	10.0 ± 2.5	.520
TAPSE (mm)	17.5 ± 3.5	18.2 ± 5.9	18.2 ± 3.9	.983

Continuous variables are expressed as mean ± SD unless indicated otherwise.

ACEI = angiotensin conversion enzyme inhibitor; ARB = angiotensin receptor blocker; ARNI = angiotensin receptor neprilysin inhibitor; CRT-D = cardiac resynchronization therapy-defibrillator; CRT-P = cardiac resynchronization therapy-pacemaker; eGFR = estimated glomerular filtration rate; LA = left atrium; LBBB = left bundle branch block; LVEF = left ventricular ejection fraction; LVESV = left ventricular end-systolic volume; MRA = mineralocorticoid receptor antagonist; NT-pro-BNP = N-terminal pro-B-type natriuretic peptide; NYHA = New York Heart Association; S’ = tricuspid annular peak systolic velocity; TAPSE = tricuspid annular plane systolic excursion.

† Available information for 144 patients.

### Patients submitted to upgrade

Table 2 presents a detailed characterization of upgraded patients, including information prior to implantation of the original device. Fifty-six patients were submitted to upgrade: 44 (78.6%) previously had a pacemaker, and 12 (21.4%) had an ICD.

**Table 2:** Characteristics of the upgraded population

	All 56 (100.0%)	Pacemaker 44 (78.6%)	ICD 12 (21.4%)	*P* value
Age (years)	70.0 ± 9.6	72.1 ± 8.3	62.4 ± 10.6	.001
Male sex, n (%)	45 (80.4%)	35 (79.5%)	10 (83.3%)	.770
Indication for pacemaker implantation, n (%)				
Sinus node dysfunction	6 (15.4%)	6 (15.4%)		
High-grade atrioventricular block	23 (66.7%)	23 (66.7%)	--	--
AF with slow ventricular response	4 (10.3%)	4 (10.3%)		
Other	3 (7.8%)	3 (7.8%)		
Pacing mode, n (%)				
DDD/DDR	26 (70.3%)	26 (70.3%)	--	--
VDD/VVIR	11 (29.7%)	11 (29.7%)		
Indication for ICD implantation, n (%)				
Primary prevention	7 (58.3%)	--	7 (58.3%)	--
Time from first implant to upgrade (years)	5.1 ± 3.3	5.9 ± 4.6	9.1 ± 3.1	.016
Ventricular stimulation (%)	73.2 ± 39.4	83.1 ± 32.1	21.9 ± 39.1	<.001
LV function prior to first device implantation, n (%)				
Preserved	16 (33.3%)	16 (44.4%)	0 (0)	.013
Reduced	32 (66.7%)	20 (54.6%)	12 (100.0%)	
LVEF prior to first device implantation (%)	38.9 ± 12.5	42.5 ± 11.8	27.9 ± 6.8	.001
HF prior to first device implantation, n (%)	20 (41.7%)	13 (36.1%)	7 (58.3%)	.176
QRS duration prior to first device implantation (ms)	130.5 ± 28.1	130.5 ± 29.2	130.3 ± 26.3	.985
LBBB prior to first device implantation, n (%)	11 (27.5%)	6 (13.6%)	4 (33.3%)	.248
Indication for upgrade, n (%)				<.001
High rate of ventricular stimulation	14 (29.4%)	11 (28.2%)	4 (33.3%)	
Pacemaker-induced LV dysfunction	25 (49.0%)	25 (64.1%)	0 (0)	
De novo LBBB	11 (21.6%)	3 (7.7%)	8 (66.7%)	
QRS duration prior to upgrade (ms)	185.2 ± 25.1	189.5 ± 23.2	169.9 ± 26.6	.015
LVEF prior to upgrade (%)	28.0 ± 6.4	29.4 ± 6.2	22.7 ± 4.0	.001
LVESV prior to upgrade (mm)	165.1 ± 78.0	138.9 ± 54.9	248.1 ± 84.2	<.001
NT-pro-BNP prior to upgrade (pg/mL)	7444.0 ± 11,946.9	6768.7 ± 9739	9216.9 ± 17,174.7	.631

Continuous variables are expressed as mean ± SD unless indicated otherwise.

HF = heart failure; ICD = implantable cardioverter-defibrillator; LBBB = left bundle branch block; LV = left ventricle; LVEF = left ventricular ejection fraction; NT-pro-BNP = N-terminal pro-B-type natriuretic peptide.

Patients upgraded from a previous pacemaker were older than ICD carriers (72.1 ± 8.3 vs 62.4 ± 10.6 years, *P* = .001). Mean duration of RV pacing previous to upgrade was 5.9 ± 4.6 years, with a mean percentage of pacemaker stimulation of 83.1% ± 32.1%. The main reason for upgrade among this group was pacemaker-induced LV dysfunction (64.1%).

Patients with prior ICDs were mostly implanted for primary prevention of SCD (58.3%). The main indication for upgrade in this group was de novo LBBB (66.7%), and all patients received a CRT-D. At the time of upgrade, patients with a previous ICD had larger LVESV (138.9 ± 54.9 mL vs 248.1 ± 84.2 mL, *P* < .001) and lower LVEF values (29.4% ± 6.2% vs 22.7% ± 4.0%, *P* = .001). A longer time elapsed between ICD implantation and upgrade to CRT, compared to pacemaker carriers (5.9 ± 4.6 years vs 9.1 ± 3.1 years, *P* = .016).

### Procedural aspects and device-related complications

A successful device implantation at first attempt was accomplished in 279 (94.6%) of patients. Procedural success was similar between groups (96.4% for upgrade vs 94.1% for de novo, *P* = .725). Epicardial lead placement was required in 9 patients (3.1%), all part of the de novo group. Concomitant AV node ablation was performed in 10 (6.6%) patients.

There were no differences regarding adverse events between upgrade and de novo groups. Lead complications (phrenic nerve stimulation, lead dislodgement or failure) were observed in 5 (8.9%) upgraded patients and in 20 patients (8.4%) receiving de novo CRT (*P* = .892). The long-term rate of device infection was also similar among both groups (1.8% vs 2.9%, *P* = .986), as was the need for device extraction (1.8% vs 2.1%, *P* = .884).

### Response to CRT

**Figure 1:**
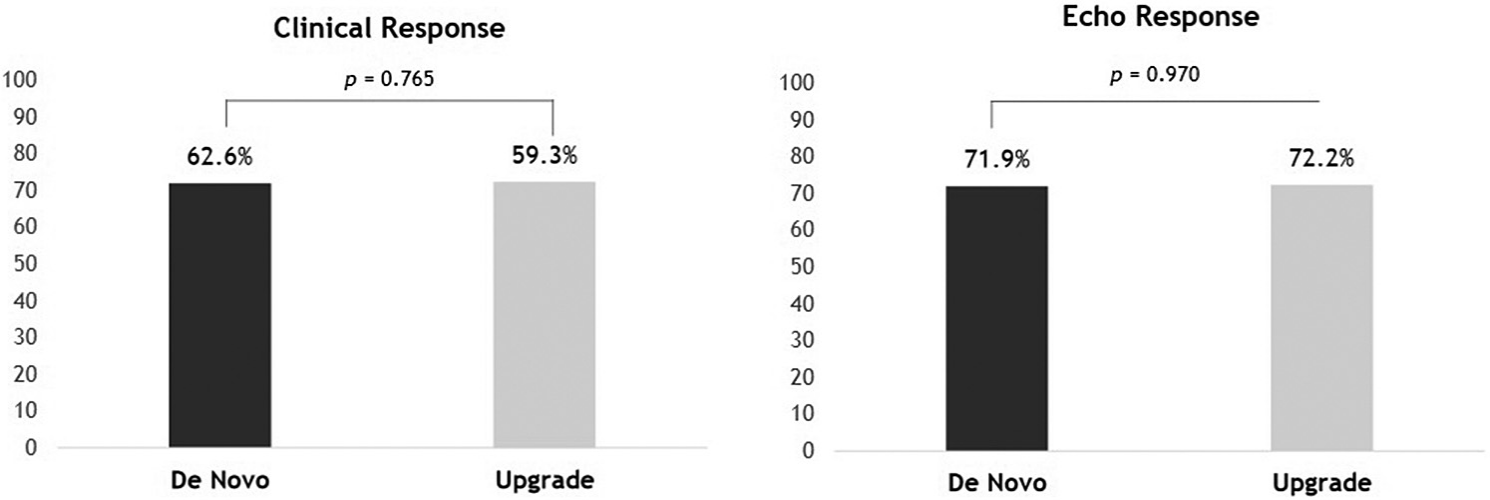
Clinical and echocardiographic response rates after de novo and upgrade to cardiac resynchronization therapy.

After an upgrade procedure, 46 (86.8%) of patients experienced functional status improvement by at least 1 NYHA class, compared with 170 (77.3%) patients in the de novo group (*P* = .179). Clinical response (Figure 1) was similar between groups (59.3% vs 62.6%, *P* = .765). The rate of super-response did not differ between groups (17.6% vs 22.2%, *P* = .472).

Echocardiographic response (Figure 1) was also comparable (72.2% vs 71.9%, *P* = .970). Similar improvements were observed in LVEF (*P* = .877) and mitral regurgitation (*P* = .121), as well as in LVESV reduction at 1 year (*P* = .684). Follow-up data are summarized in Table 3.

**Table 3:** Follow-up data at 1-year post cardiac resynchronization therapy implant

Variable	All (N = 295)	De novo (N = 239)	Upgrade (N = 56)	*P* value
NYHA class improvement, n (%)	216 (79.1%)	170 (77.3%)	46 (86.8%)	.179
NYHA class, n (%)				
I/II	226 (94.6%)	182 (94.3%)	44 (95.7%)	.999
III/IV	13 (5.4%)	11 (5.7%)	2 (4.3%)	
Clinical response, n (%)	171 (62.0%)	139 (62.6%)	32 (59.3%)	.765
HHF, n (%)	51 (19.2%)	37 (17.1%)	14 (28.6%)	.099
Ventricular arrhythmias, n (%)	18 (6.9%)	15 (7.1%)	3 (6.0%)	.775
Appropriate ICD therapies, n (%)	16 (6.2%)	14 (6.7%)	2 (4.0%)	.481
LVEF (%)	39.3 ± 10.1	39.6 ± 10.0	37.7 ± 10.5	.588
Δ LVEF (%)	11.5 ± 8.3	10.7 ± 8.8	9.6 ± 8.8	.877
Δ LVESV (mL)	-45.9 ± 49.4	-46.1 ± 50.1	-45.5 ± 47.5	.684
Degree of MR, n (%)				.494
Mild	117 (51.5%)	96 (51.3%)	21 (52.5%)	
Mild-to-moderate	66 (29.1%)	57 (30.5%)	9 (22.5%)	
Moderate-to-severe	35 (15.4%)	28 (15.0%)	7 (17.5%)	
Severe	9 (4.0%)	6 (3.2%)	3 (7.5%)	
Echo response, n (%)	113 (72.0%)	87 (71.9%)	26 (72.2%)	.970
Super-response, n (%)	59 (21.4%)	50 (22.2%)	9 (17.6%)	.472
MACE, n (%)	102 (36.2%)	81 (35.5%)	21 (39.6%)	.689
All-cause mortality, n (%)	73 (24.7%)	62 (25.9%)	11 (19.6%)	.417

Continuous variables are expressed as mean ± SD unless indicated otherwise.

HHF = hospitalization for heart failure; ICD = implantable cardioverter-defibrillator; LVEF = left ventricular ejection fraction; LVESV = left ventricular end-systolic volume; MACE = major adverse cardiac events; MR = mitral regurgitation; NT-pro-BNP = N-terminal pro-B-type natriuretic peptide; NYHA = New York Heart Association; S’ = tricuspid annular peak systolic velocity.

### Long-term follow-up and survival

In the overall population, during a median follow-up period of 3 years (interquartile range 1–6 years, maximum period 16 years), 73 (24.7%) patients died: 11 (19.6%) in the upgrade group and 62 (25.9%) in the de novo group (*P* = .417). Fourteen (28.6%) patients from the upgrade group and 37 (17.1%) from the de novo group were hospitalized for HF within the first year after CRT implantation (*P* = .099).

**Figure 2:**
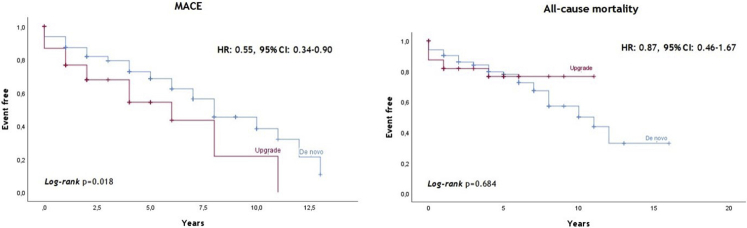
Clinical outcomes of the overall population. CI = confidence interval; HR = hazard ratio; MACE = major adverse cardiac events.

In Kaplan-Meier survival analysis (Figure 2), all-cause mortality was similar among groups (*P* = .684), but MACE occurred less frequently in the de novo group (hazard ratio [HR]: 0.55, 95% confidence interval [CI]: 0.34–0.90, *P* = .018).

In the PSM analysis, a cohort of 106 matched pairs (56 upgrade and 50 de novo patients) without baseline statistical differences was assembled. Baseline characteristics of the propensity-matched cohort are depicted in Supplemental Table 1.

In this cohort, the rate of hospitalization for HF during the first year of follow-up was similar between upgraded and de novo patients (28.6% vs 27.7%, *P* = .921). Mortality during follow-up was also comparable (19.6% vs 30.0%, *P* = .216).

**Figure 3:**
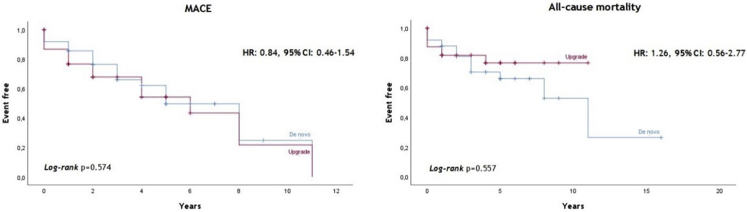
Clinical outcomes of the propensity score–matched cohort. CI = confidence interval; HR = hazard ratio; MACE = major adverse cardiac events.

In the propensity-matched samples, all-cause mortality (HR: 1.26, 95% CI: 0.56–2.77, *P* = .557) and MACE (HR: 0.84, 95% CI: 0.46–1.54, *P* = .574) were comparable between upgrade and de novo patients (Figure 3).

## Discussion

### Main findings

The main finding of our study, including nearly 300 CRT patients managed in a single center, is that survival after upgrade to resynchronization therapy was comparable to de novo implants. In accordance, clinical and echocardiographic response to CRT in upgraded patients was similar to de novo patients. These results suggest that upgrade to CRT may confer morbidity and mortality benefits to HF patients, as previously shown in randomized trials with de novo device recipients.

### Patients submitted to upgrade

The majority of upgraded patients previously had a pacemaker, and subsequently developed pacemaker-induced LV dysfunction. At the time of the upgrade procedure, these subjects were on average 10 years older than ICD carriers; naturally, they presented larger QRS and a higher percentage of ventricular stimulation.

Patients from the ICD group were upgraded, on average, nearly 10 years after implantation of the first device. As expected, ICD recipients presented features of more advanced HF, such as lower LVEF and larger LVESV. The main reason for upgrade was new-onset LBBB. In cases of longstanding HF, a superimposed LBBB may be a marker of disease progression, with possible implications in the response to resynchronization. This may explain the heterogeneity of outcomes and the opposing results from previous observational studies.

### Procedural aspects and device-related complications

Upgrade to CRT was believed to carry a higher risk of procedural and short-term complications. The REPLACE registry^12^ raised concerns regarding the safety of upgrade procedures. In this study analyzing complication rates in patients undergoing generator replacement, with or without lead addition, the highest risk of major complications was observed after procedures to add an LV lead for CRT.^12^ The RAFT upgrade substudy, contrastingly, showed a higher proportion of acute complications with de novo CRT implants compared to upgrades.^13^ The European CRT Survey provided some reassurance, since upgrade procedures presented a similar complication rate to de novo implants.^5^ In a recently published large study, procedure-related morbidity was comparable in patients who underwent CRT upgrade or generator replacement only.^14^ Lead dislodgement and other mechanical complications were, however, more frequent in the upgrade cohort.^14^ More recently, a study from a high-volume American center compared procedural outcomes between de novo and upgrade to CRT, and found no difference in the rate of procedural success or 90-day complications between groups.^15^

In line with these studies, our analysis did not show differences in lead complications or device-related infection between upgrade and de novo implants.

Importantly, in our center, coronary sinus cannulation is performed using electrophysiology catheters and electrogram guidance. This strategy is associated with a decrease in the use of contrast, fluoroscopy, and total procedure time.^16^

### Response to CRT

In our study, clinical and echocardiographic response to CRT were comparable in upgraded and de novo patients. A low incidence of ventricular arrhythmias was observed in either group. Of note, patients from both groups were under optimal treatment with guideline-directed therapy: a large percentage of patients in our study were previously treated with a mineralocorticoid receptor antagonist (56.4% in the overall population, 67.9% in the upgrade group), a percentage higher than observed in the reviewed literature.^11^

Previous studies showed worse outcomes in upgraded patients than in those undergoing de novo implants.^8^^,^^17^^,^^18^ Vamos and colleagues^8^ found NYHA class improvement to be less common after upgrade. Also, in the European CRT Survey, more patients submitted to upgrade reported unchanged global assessment status.^5^ It has been hypothesized that the lack of benefit with upgrade results from procedures being performed too late, in patients submitted to several years of detrimental RV pacing. However, Fröhlich and colleagues^10^ observed significant improvements in LVEF and reverse remodeling in upgraded patients, even after very long periods (up to 10 years) of RV pacing. In fact, in our cohort, 25.6% (13) of our upgraded patients were under RV pacing for more than 10 years; the longest exposure was of 17 years in 1 patient.

### Long-term follow-up and survival

In our study, survival after upgrade to resynchronization therapy was comparable to de novo implants. A multicenter German study, enrolling 552 patients, found survival after upgrade to be significantly worse than with de novo CRT.^8^ All-cause mortality remained significantly higher in the upgrade group, even after adjusting for potential confounders and PSM.^8^ Cheung and colleagues,^17^ in an analysis of a nationwide American database, reported significantly higher rates of adverse postprocedural outcomes with CRT upgrades. Moreover, upgrades were independently associated with a 2-fold increase in in-hospital mortality compared to de novo CRT implants.^16^

In the European CRT Survey, 1-year mortality was similar in de novo patients and in patients with previous devices.^5^ Leyva and colleagues described a similar long-term risk of mortality and hospitalization for HF in patients with preexisting pacemakers and patients undergoing de novo CRT.^11^ Recently, a large American registry comprising thousands of patients with pre-existing ICDs upgraded to CRT showed a significant reduction in mortality at 3-year follow-up after upgrade, compared to eligible patients who underwent solely generator replacement.^14^ However, CRT upgrade did not impact rates of hospitalization.^14^

### Current and future perspectives

While randomized data comparing upgrade to de novo resynchronization therapy is currently lacking, evidence to guide clinical practice and support decision-making is mostly derived from the European CRT Survey^5^ and observational studies with conflicting results.

Whether previous pacemaker or ICD recipients who subsequently develop indications for resynchronization should be upgraded to a CRT device is still debatable. Our study, and some previous registries, suggest nonetheless that the benefit with CRT observed in the de novo trials may extend to patients with previous devices who later become eligible for resynchronization.

However, eligibility criteria, patient selection, and optimal timing for upgrade remain a challenge for HF teams. The ongoing BUDAPEST-CRT Upgrade Study [NCT02270840],^19^ designed to randomize patients with previous pacemaker/ICD to receive a CRT-D or an ICD, will, it is hoped, provide further insight on the benefits and harms of upgrade to CRT.

### Limitations and strengths

There are several limitations to our study. Given its retrospective nature, data collection and complete retrieval of information was occasionally challenging, especially in patients referred from other institutions and the deceased.

Unfortunately, we did not report echocardiographic parameters indexed to body surface area, since anthropometric data were unavailable in some patients. Also, HF symptoms were only subjectively assessed and translated to the NYHA classification; no questionnaires or scores were applied.

Nevertheless, we presented a detailed characterization of upgraded patients, including information dating back to the time of the previous device implantation. To our knowledge, this is the first study analyzing clinical outcomes after upgrade to extensively report these data.

Also, we provide one of the longest follow-up durations after upgrade reported to date. Therefore, our study fills a gap found in previous studies and may, despite its limitations, enlighten and aid the decision-making process surrounding CRT upgrade.

## Conclusion

In our cohort of almost 300 CRT patients, survival after upgrade to resynchronization therapy was comparable to de novo implants. Despite their older age and a higher comorbidity burden, upgraded patients exhibited similar clinical and echocardiographic response to de novo patients. The results of our study suggest that upgrade to CRT may confer morbidity and mortality benefits to HF patients, as previously shown in randomized trials with de novo device recipients. The ongoing BUDAPEST-CRT trial will provide the first randomized evidence in this area and, we hope, help guide clinical practice regarding upgrade to CRT.

## Funding Sources

This research did not receive any specific grant from funding agencies in the public, commercial, or not-for-profit sectors.

## Disclosures

The authors have no conflicts to disclose.

## Authorship

All authors attest they meet the current ICMJE criteria for authorship.

## Patient Consent

Patients provided informed consent for data collection in each evaluation.

## Ethics Statement

The study was conducted in accordance with the Declaration of Helsinki and was approved by the local Ethics Committee.
