# Supplementary material for: Long-term clinical outcomes after upgrade to resynchronization therapy: A propensity score–matched analysis
Source: Heart Rhythm O2. 2021 Dec 17;2(6Part B):671–9. doi: 10.1016/j.hroo.2021.06.009 (PMC8710617; doi:10.1016/j.hroo.2021.06.009)
Supplement: Supplemental Table 1 [file mmc1.docx]

**Supplemental Table 1: Characterization of the propensity-score matched cohort**

| **Variable** | **All**  (N=106) | **De novo**  (N=50) | **Upgrade**  (N=56) | *p-*value |
| --- | --- | --- | --- | --- |
| Male, n (%) | 84 (79.2%) | 39 (78.0%) | 45 (80.4%) | 0.953 |
| Age (years) | 69.5 ± 10.5 | 68.5 ± 11.5 | 70.0 ± 9.6 | 0.128 |
| Device type, n (%)  CRT-P  CRT-D | 68 (64.2%) 38 (35.8%) | 28 (56.0%)  22 (44.0%) | 40 (71.4%)  16 (28.6%) | 0.147 |
| Primary prevention, n (%) | 22 (61.1%) | 15 (71.4%) | 7 (46.7%) | 0.248 |
| Type of cardiomyopathy, n (%)  Ischemic  Non-ischemic | 38 (36.2%)  67 (63.8%) | 18 (32.1%)  29 (59.2%) | 20 (40.8%)  38 (67.9%) | 0.472 |
| Baseline NYHA class | 2.9 ± 0.6 | 2.8 ± 0.6 | 2.9 ± 0.6 | 0.922 |
| Arterial hypertension, n (%) | 79 (75.2%) | 37 (74.0%) | 42 (76.4%) | 0.957 |
| Diabetes mellitus, n (%) | 43 (41.0%) | 19 (38.0%) | 24 (43.6%) | 0.698 |
| Coronary artery disease, n (%) | 45 (42.9%) | 22 (44.0%) | 23 (41.8%) | 0.977 |
| Atrial fibrillation, n (%) | 54 (51.4%) | 25 (50.0%) | 29 (52.7%) | 0.933 |
| Valvular heart disease  (moderate to severe) , n (%) | 46 (43.4%) | 22 (44.0%) | 24 (42.9%) | 0.906 |
| Chronic kidney disease (eGFR<60 ml/min/1.73m^2^), n (%) | 37 (35.2%) | 17 (34.0%) | 20 (36.4%) | 0.961 |
| Use of ACEI/ARB/ARNI, n (%) | 83 (80.6%) | 40 (80.0%) | 43 (81.1%) | 0.885 |
| Use of beta-blockers, n (%) | 84 (81.6%) | 38 (76.0%) | 46 (86.8%) | 0.247 |
| Use of MRA, n (%) | 158 (56.4%) | 40 (52.6%) | 36 (47.4%) | 0.243 |
| Use of loop diuretic, n (%) | 90 (89.1%) | 44 (88.0%) | 46 (90.2%) | 0.972 |
| LBBB or paced QRS, n (%) | 104 (98.1%) | 49 (98.0%) | 55 (98.2%) | 0.931 |
| QRS duration (ms) | 177.5 ± 25.0 | 164.3 ± 32.1 | 185.2 ± 25.1 | 0.943 |
| LVEF (%) | 28.7 ± 7.1 | 29.6 ± 7.7 | 28.0 ± 6.4 | 0.176 |

Continuous variables are expressed as (Mean ± SD) unless indicated otherwise.

ACEI: angiotensin conversion enzyme inhibitor. ARB: angiotensin receptor blocker. ARNI: angiotensin receptor neprilysin inhibitor. CRT-D: cardiac resynchronization therapy-defibrillator. CRT-P: cardiac resynchronization therapy-pacemaker. eGFR: estimated glomerular filtration rate. LBBB: left bundle branch block. NYHA: New York Heart Association.
